# The Na^+^, K^+^-ATPase **β**1 subunit regulates epithelial tight junctions via MRCK**α**

**DOI:** 10.1172/jci.insight.134881

**Published:** 2021-02-22

**Authors:** Haiqing Bai, Rui Zhou, Michael Barravecchia, Rosemary Norman, Alan Friedman, Deborah Yu, Xin Lin, Jennifer L. Young, David A. Dean

**Affiliations:** 1 Department of Pediatrics and; 2 Department of Pathology, School of Medicine and Dentistry, University of Rochester, Rochester, New York, USA.; 3 Department of Materials Design and Innovation, School of Engineering and Applied Sciences, University at Buffalo, Buffalo, New York, USA.

**Keywords:** Cell Biology, Pulmonology, Gene therapy, Tight junctions

## Abstract

An intact lung epithelial barrier is essential for lung homeostasis. The Na^+^, K^+^-ATPase (NKA), primarily serving as an ion transporter, also regulates epithelial barrier function via modulation of tight junctions. However, the underlying mechanism is not well understood. Here, we show that overexpression of the NKA β1 subunit upregulates the expression of tight junction proteins, leading to increased alveolar epithelial barrier function by an ion transport–independent mechanism. Using IP and mass spectrometry, we identified a number of unknown protein interactions of the β1 subunit, including a top candidate, myotonic dystrophy kinase–related cdc42-binding kinase α (MRCKα), which is a protein kinase known to regulate peripheral actin formation. Using a doxycycline-inducible gene expression system, we demonstrated that MRCKα and its downstream activation of myosin light chain is required for the regulation of alveolar barrier function by the NKA β1 subunit. Importantly, MRCKα is expressed in both human airways and alveoli and has reduced expression in patients with acute respiratory distress syndrome (ARDS), a lung illness that can be caused by multiple direct and indirect insults, including the infection of influenza virus and SARS-CoV-2. Our results have elucidated a potentially novel mechanism by which NKA regulates epithelial tight junctions and have identified potential drug targets for treating ARDS and other pulmonary diseases that are caused by barrier dysfunction.

## Graphical abstract

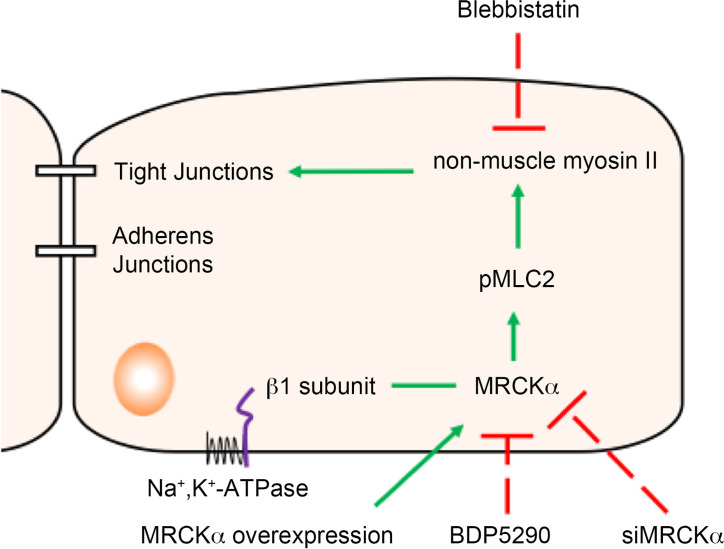

## Introduction

An intact alveolar epithelial barrier is essential for normal gas exchange and alveolar liquid reabsorption. Damage of the barrier is associated with serious lung diseases, such as acute respiratory distress syndrome (ARDS), a life-threatening condition that affects over 190,000 people each year in the United States and accounts for 74,500 deaths (1). Current therapies for ARDS rely on supportive care, rather than targeting the underlying pathophysiology of the disease, which is characterized by a leaky lung barrier. Although various mechanisms that lead to its disruption have been identified (2), pathways that may restore the normal lung barrier function remain underexplored.

The Na^+^, K^+^-ATPase (NKA) is a multifunctional transmembrane protein that is expressed in all mammalian cells. In the alveolar epithelium, it is located at the basolateral surface of alveolar epithelial type I cells (ATI) and alveolar epithelial type II cells (ATII) and produces a vectorial ion gradient that is required for fluid balance in the lung air space. The whole pump is composed of a catalytic α subunit and a noncatalytic β subunit, which facilitates maturation and membrane targeting of the α subunit. Both subunits have decreased expression and/or activity in lung illnesses resulting from respiratory viral infection or lung edema (3–6). Overexpression of these subunits in ARDS animal models accelerated alveolar fluid clearance, reduced lung edema, and improved disease outcome (7–13). Surprisingly, the noncatalytic β1 subunit was found to provide a protective role in the alveolar epithelial barrier, as demonstrated by increased expression of tight junction proteins and decreased alveolar barrier permeability (8, 13). However, whether the effect is due to pump activity or other signaling pathways remains elusive.

In this study, we aimed to identify the signaling pathway by which the NKA β1 subunit regulates alveolar tight junctions. We first demonstrated that the barrier-enhancing effect is specific to the β1 subunit and appears to be independent of the ion transport activity. Using IP and mass spectrometry (MS), we identified a number of previously unreported interacting partners of the β1 subunit. Among them, MRCKα is a serine/threonine protein kinase that may regulate the tight junction assembly pathway. Using loss-of-function, chemical inhibition, and gain-of-function experiments, we revealed a potentially novel molecular pathway by which the β1 subunit binds and activates MRCKα, thereby phosphorylating non–muscle myosin II and increasing tight junction levels at the membrane. Interestingly, the expression of MRCKα in lungs from ARDS patients is significantly decreased. Together, our findings have elucidated a molecular pathway by which NKA regulates alveolar barrier function by interacting with and activating MRCKα.

## Results

### β1 subunit overexpression increases expression of alveolar tight junctions.

**Figure 1:**
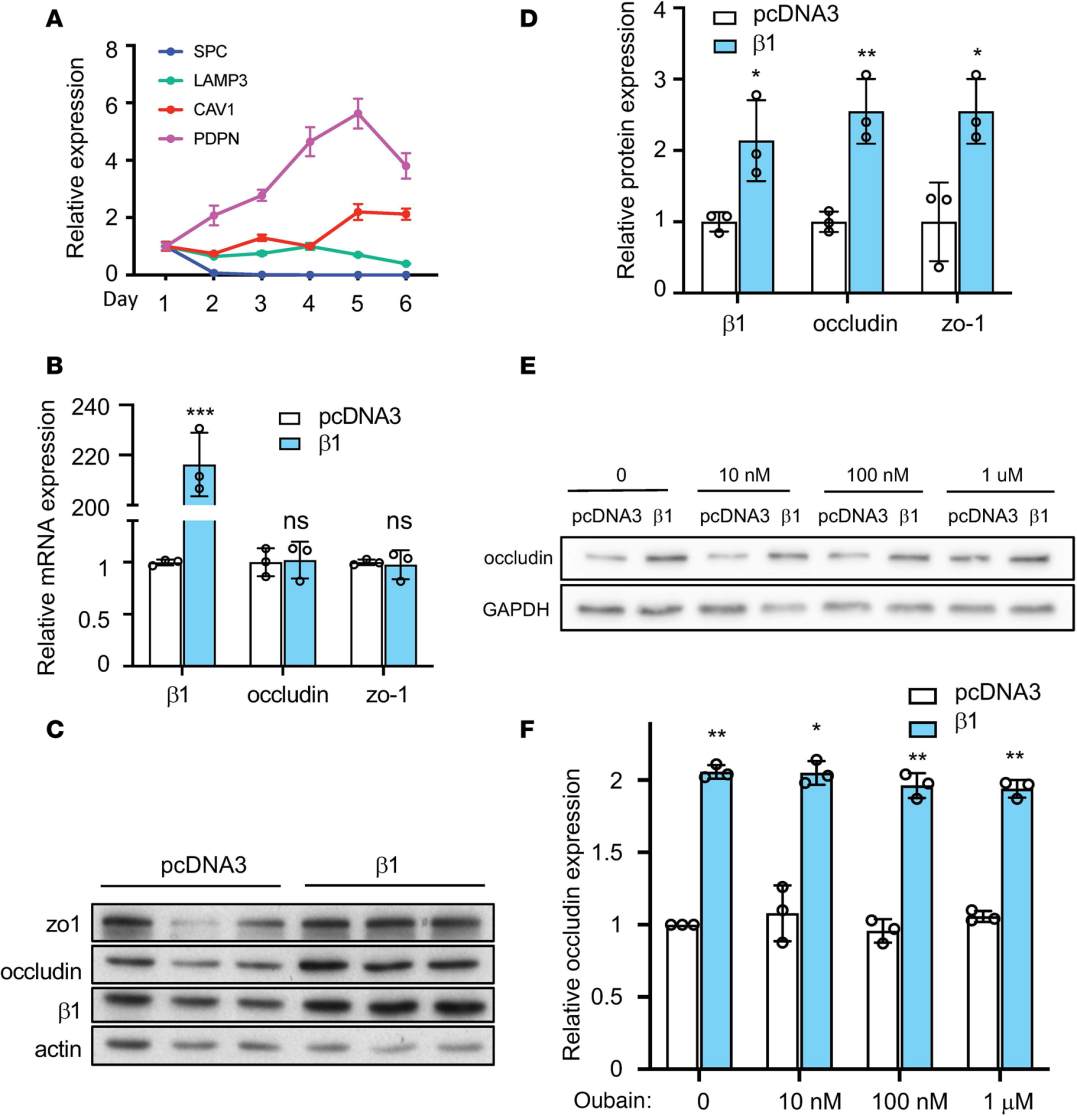
Overexpression of the β1 subunit increases expression of tight junction proteins. (**A**) Rat primary ATII cells differentiate into ATI-like cells when cultured in vitro. Cells were lysed for qPCR analysis of ATII markers (*SPC* and *LAMP3*) and ATI markers (*CAV1* and *PDPN*) at different days after culture. Data represents *n* = 3 biological replicates. (**B**) Relative mRNA levels for occludin and zo-1 in control and the β1 subunit–transfected cells. (**C**) Cells were transfected with plasmid expressing the rat β1 subunit or pCDNA3 empty plasmid as control at day 3 after isolation. Cells were lysed for Western blot 24 hours later. (**D**) Quantification of the Western blots in **C**. Data are representative of 3 independent experiments. Data are presented as mean ± SD. Statistical analysis was performed by 2-tailed Student’s *t* test. *P < 0.05; **P < 0.01; ***P < 0.001. (**E**) Ouabain treatment at the indicated concentrations does not inhibit the β1 subunit–mediated occludin upregulation. AT1 cells were transfected with the pCDNA3 plasmid or the pCMV-β1 plasmid, and 4 hours later, the DMSO control or ouabain at 10 nM, 100 nM, or 1 μM was added to cells. (**F**) Western blots were performed at 24 hours after transfection and were quantified. Data are representative of 3 independent experiments. Data are presented as mean ± SD. Statistical analysis was performed by 2-tailed Student’s *t* test. *P < 0.05; **P < 0.01.

Rat primary alveolar epithelial cells were used to investigate the role of the β1 subunit on tight junctions. To characterize these cells, quantitative PCR (qPCR) analysis was performed for genes that are specific for ATI (*CAV1* and *PDPN*) or ATII (*SPC* and *LAMP3*) cells (Figure 1A). *SPC* and *LAMP3* levels were high at 24 hours after isolation but decreased after 72 hours while *CAV1* and PDPN levels gradually arise, suggesting differentiation from an ATII phenotype to an ATI phenotype. When cultured in transwell plates coated with 20 μg/mL fibronectin, these cells developed high transepithelial electrical resistance (TEER) (Supplemental Figure 1A; supplemental material available online with this article; https://doi.org/10.1172/jci.insight.134881DS1) and displayed membrane staining of occludin and zo-1 (Supplemental Figure 1B). In addition, when treated with 1 μg/mL LPS, a bacterial endotoxin, these cells showed decreased TEER and increased permeability to 4 kD dextran (Supplemental Figure 1, C and D), indicating barrier damage. Thus, the rat primary ATI culture system can serve as a relevant model to study the alveolar epithelial barrier.

Next, we aimed to overexpress the β1 subunit in ATI cells in order to examine its role in epithelial barrier function. We used electroporation to transfect ATI cells with a plasmid encoding the rat β1 subunit, and we measured expression of tight junction proteins 24 hours later. Transfection of the β1 subunit had no effect on the mRNA abundance of occludin or zo-1 (Figure 1B). However, at the protein level, we observed significant increases of both proteins (Figure 1, C and D). We also observed increased levels of zo-2 and claudin-18 (Supplemental Figure 1E), a tight junction component that recently has been found to play an essential role in alveolar barrier properties (14–17). Notably, treatment with the NKA-specific inhibitor ouabain at concentrations that block the enzymatic and ion transport activity of the NKA α subunit (18) failed to block the upregulation of occludin (Figure 1, E and F). In parallel, when cells were transfected with the β2 or the β3 subunit, 2 other NKA β isoforms that can also form functional heterodimers with the α subunit, no upregulation of tight junction proteins was observed (Supplemental Figure 2, A and B). These data demonstrate that overexpression of the β1 subunit of the NKA increases the expression of many tight junction proteins, and this effect is independent of its regulatory role in pump activity of NKA αβ heterodimers.

### Overexpression of the β1 subunit increases ATI barrier integrity.

**Figure 2:**
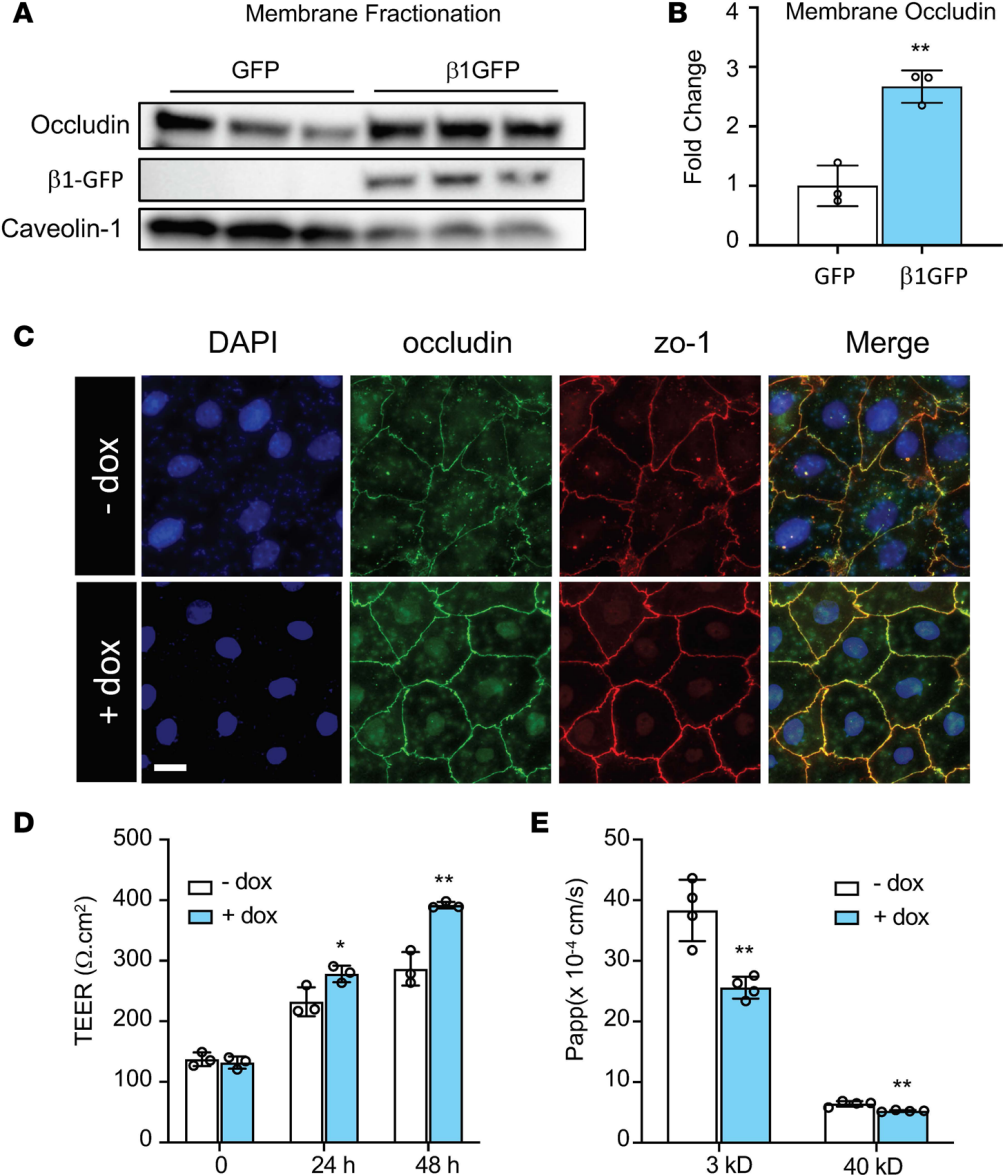
Overexpression of the β1 subunit increases alveolar type I barrier function. (**A**) Overexpression of the β1 subunit in HEK293T cells increased occludin expression at the plasma membrane. Caveolin-1 was used as membrane loading control. (**B**) Densitometry of gels in **A**, with analysis by Student’s *t* test, **P < 0.01. (**C**) ATII cells were cotransfected with 4 mg/mL pCMV-Tet3G plasmid and 16 mg/mL pTet3G-human β1 plasmid day 1 after isolation. Cells were then plated on fibronectin-coated coverslips. Doxycycline (1 μg/mL) was added 48 hours later. Representative immunofluorescence staining of ATI cells shows that doxycycline-induced expression of the β1 subunit induces more mature tight junctions, as indicated by occludin (green) and zo-1 (red) staining. Images represent 3 independent experiments. Scale bar: 20 mm. (**D**) ATII cells were cotransfected as in **C**, but cells were plated on fibronectin-coated 12-well transwell plates. Twenty-four hours later at day 2, 1 μg/mL of doxycycline (dox) was added to induce β1 gene expression. TEER was measured every 24 hours. ANOVA followed by Bonferroni’s post hoc test was used for statistical analysis, *P < 0.05, **P < 0.01. (**E**) After TEER measurement at day 4, permeability to 3 kD Texas Red–dextran and 40 kD FITC-dextran was measured for a duration of 2 hours. Data are presented as mean ± SD. ANOVA followed by Bonferroni’s post hoc test was used for statistical analysis, **P < 0.01.

Given that tight junctions components are localized to the apical membrane of cells in a mature epithelial layer, we aimed to investigate if the β1 subunit increases tight junctions at cell membranes and enhances alveolar barrier function. Western blot of the plasma membrane fraction shows that the β1 subunit significantly increases the level of membrane-associated occludin (Figure 2, A and B). To explore the relevance to barrier function, we developed a doxycycline-inducible system to control β1 subunit expression by cloning the rat β1 subunit into a Tet-on plasmid. To characterize the system, human bronchial epithelial cells 16HBE14o- were cotransfected with pCMV-tet regulator plasmids and pTet3G-human β1 subunit expressing plasmids, followed immediately by addition of doxycycline (0, 1, 10, 100, and 1000 ng/mL). Immunoblot analysis showed that doxycycline caused a dose-dependent upregulation of the β1 subunit at 24 hours after transfection with maximal induction at 1000 ng/mL (Supplemental Figure 3A). Consequently, the expression of occludin and zo-1 were also increased proportionally, confirming previous findings using the pCMV-rat β1 plasmid. Cotransfection of the regulator plasmid and a luciferase reporter plasmid confirmed robust transgene expression for up to 7 days after transfection (Supplemental Figure 3B). qPCR for SPC and Cav1 showed that doxycycline does not affect the differentiation from ATII to ATI (Supplemental Figure 3C). Next, we sought to examine the role of the β1 subunit in epithelial barrier function. Immunostaining at 48 hours after induction of β1 transgene expression showed increased localization of occludin and zo-1 at cell-to-cell junctions, indicating more mature epithelial barriers than controls (Figure 2C). TEER was significantly higher at 24 hours and 48 hours after doxycycline induction (Figure 2D), suggesting an increase in barrier integrity. In accordance, the permeabilities to 3 kD dextran and 40 kD dextran decreased by 33.2% and 18.5%, respectively, following doxycycline treatment for 48 hours (Figure 2E). Taken together, our data demonstrate that overexpression of the NKA β1 subunit leads to improved alveolar epithelial barrier function.

### Identification of the β1 subunit interactome.

**Figure 3:**
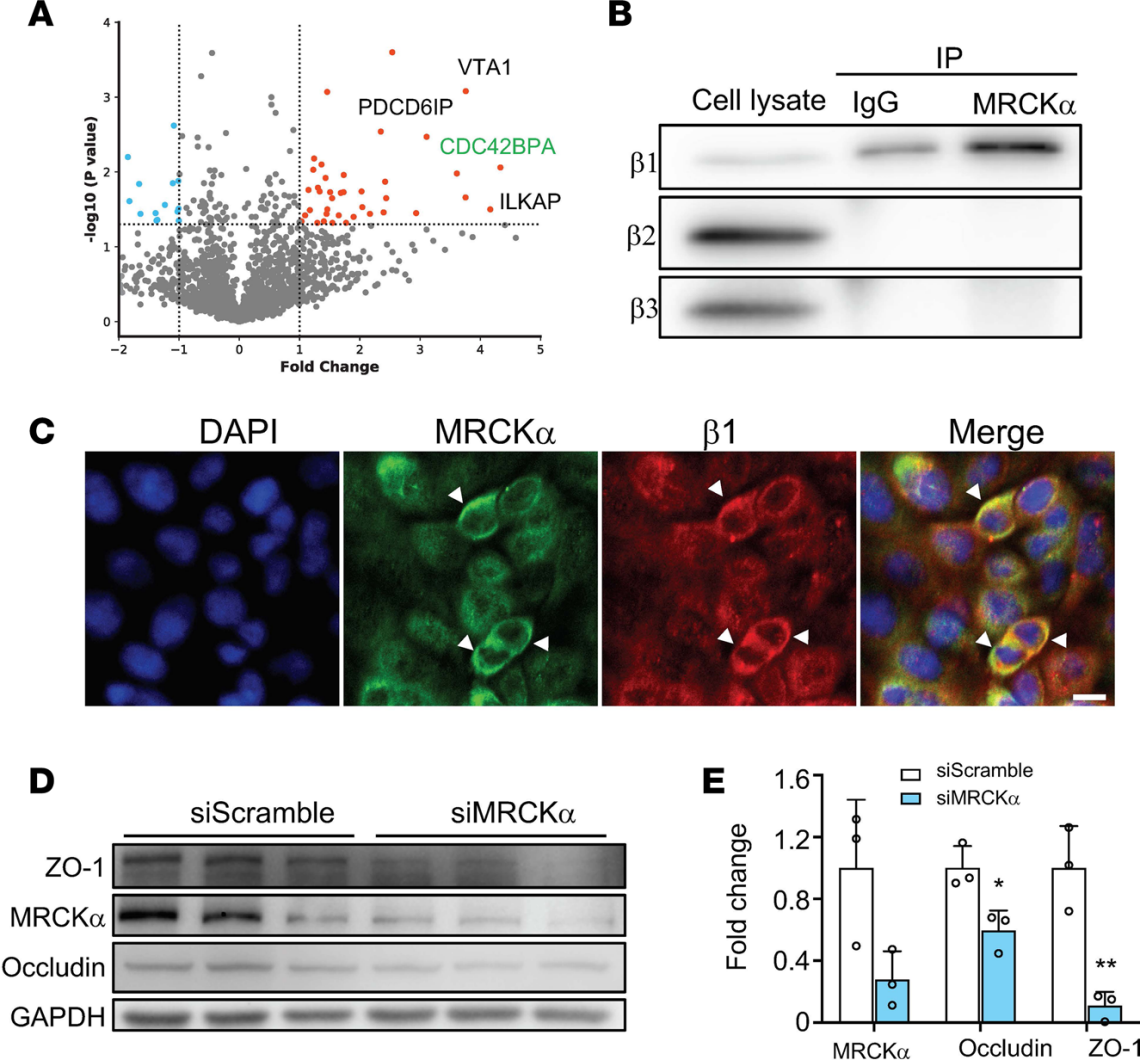
MRCKα interacts with the β1 subunit and stabilizes tight junction. (**A**) Volcano plot of proteins identified from triplicate mass spectrometry experiments. CDC42BPA (MRCKα) is labeled on the graph. Dashed line indicates the *P* value threshold of 0.05. (**B**) The interaction of MRCKα with the β1 subunit was confirmed using co-IP. A total of 5% of total cell lysate was used for input. The β2 or β3 subunit did not coimmunoprecipitate with MRCKα. (**C**) The β1 subunit (red) and MRCKα (green) colocalize in ATI cells. Scale bar: 20 μm. (**D**) ATI cells were transfected with a scrambled siRNA (siScramble) or a siRNA against MRCKα (siMRCKα). (**E**) Twenty-four hours later, cells were lysed for immunoblot analysis and quantified. Data represent 3 biological replicates and Error bars show SD. Student’s *t* test, *P < 0.05, **P < 0.01.

Since our findings suggest that the β1 subunit–mediated epithelial barrier tightening is independent of its role in ion transport activity of the NKA, we reasoned that this regulation may act through protein-to-protein interactions. So far, a limited number of protein interactions of the β1 subunit have been reported in the literature. To overcome this, we used MS to systematically identify binding partners of the β1 subunit. Cell lysates from untransfected 16HBE14o- cells were immunoprecipitated using an antibody against the β1 subunit or an antibody against GFP as a negative control. The resulting protein complexes were separated by SDS-PAGE, and the gels were cut into 10 segments to increase resolution of protein identification. The proteins were extracted from the gel segments and subjected to trypsin digestion (Supplemental Figure 4A). After database searching for the spectrums, we identified 2936 unique proteins from 3 independent experiments (Supplemental Table 1). We then quantified their relative abundance using normalized spectrum abundance factor (NSAF), a label-free quantification method based on counting the number of unique peptides assigned to each protein (19). A total of 138 proteins passed the criteria for potential interactions (*P* < 0.05, Student’s *t* test) (Figure 3A). Top candidates include *CDC42BPA* (serine/threonine-protein kinase MRCKα), *ILKAP* (integrin-linked kinase–associated serine/threonine phosphatase 2), and *VTA1* (vacuolar protein sorting–associated protein VTA1 homolog) (Table 1). Gene Ontology (GO) enrichment analysis (20) of the identified protein interactors revealed significant enrichment for biological processes, including endosomal sorting complex required for transport (ESCRT) disassembly and multivesicular body organization, 2 processes involved in the endosomal sorting of ubiquitylated membrane proteins (Table 2).

### MRCKα regulates epithelial barrier integrity.

One of the top proteins identified from our MS experiment is MRCKα, a serine/threonine-protein kinase and a downstream effector of Cdc42 in cytoskeletal reorganization. In its native state, MRCKα forms a homodimer that blocks its kinase activity (21). Once activated, it phosphorylates substrates including myosin light chain 2 (MLC2) and LIM kinase, thereby modulating actin-myosin contraction (22). The dissociation of the autoinhibitory homodimerization complex is a prerequisite for MRCKα activation, which can be induced by a number of factors, such as Rap1 (23) and PDK1 (24). By regulating the cytoskeleton, activated MRCKα is involved in many cellular processes, such as cell migration (24, 25), cell polarity (26), and endothelial junction formation (23, 27). We hypothesized that the β1 subunit may increase alveolar epithelial barrier integrity through MRCKα.

MRCKα had more than 40% sequence coverage from our MS analysis (Supplemental Figure 4B). To further confirm its interaction with the β1 subunit, we performed a co-IP experiment in untransfected 16HBE14o- cells. Among the 3 endogenous β isoforms, only the β1 subunit was detected in the MRCKα pulldown complex, suggesting the specificity of the interaction (Figure 3B). Further immunofluorescence staining in ATI cells showed that β1 colocalizes with MRCKα on the cell membrane (Figure 3C). To investigate the functional role of MRCKα in the epithelial barrier, we knocked down its expression in ATI cells and evaluated protein levels of tight junctions. Cells transfected with small interfering RNA (siRNA) against MRCKα showed significantly lower levels of both occludin and zo-1 (Figure 3, D and E), suggesting that MRCKα may stabilize the expression of tight junction proteins.

**Figure 4:**
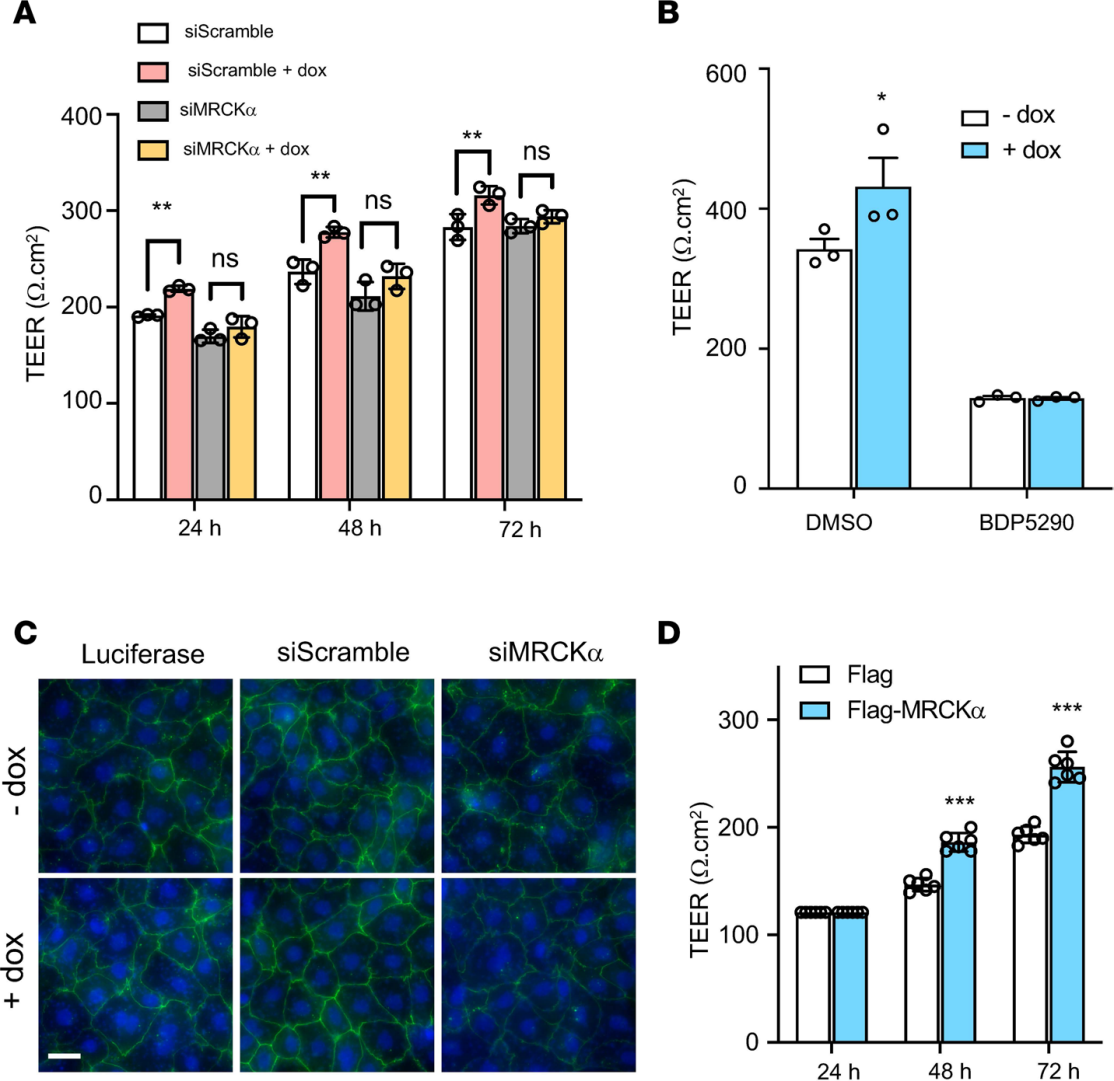
MRCKα is required for the β1 subunit–mediated alveolar barrier tightening. (**A**) ATII cells were cotransfected with siRNA (scramble control or against MRCKα) and plasmids (CMV-tet and Tet- β1) 24 hours after isolation. A total of 1 μg/mL doxycycline was added to induce gene expression at day 2. TEER was then measured every 24 hours from day 3 to day 5. ANOVA followed by Bonferroni’s post hoc test was used for statistical analysis, **P < 0.01. (**B**) ATII cells were cotransfected with plasmids (CMV-tet and Tet-β1) 24 hours after isolation and treated immediately with 2 μM MRCKα inhibitor BDP5290. TEER was measured 24 hours later. Data are presented as mean ± SD. ANOVA followed by Bonferroni’s post hoc test was used for statistical analysis, *P < 0.05. (**C**) Immunofluorescence staining of zo-1 in cells treated with or without doxycycline for 48 hours after transfection with luciferase plasmid alone, β1 plasmid and siScramble, or β1 plasmid and siMRCKα. Images represent 3 separate experiments. Scale bar: 20 um.(**D**) Overexpression of MRCKα increases TEER. Data represent *n* = 6 biological replicates. Data are presented as mean ± SD. ANOVA followed by Bonferroni’s post hoc test was used for statistical analysis, ***P < 0.001.

Since MRCKα loss of function impairs tight junctions, we hypothesized that the NKA β1 subunit enhances alveolar barrier function through its interaction with MRCKα. To test this hypothesis, we first depleted MRCKα using siRNA and subsequently induced β1 overexpression using doxycycline. TEER was significantly higher in ATI monolayers at 24, 48, and 72 hours after doxycycline treatment, but it was abolished when cells were transfected with siRNA against MRCKα (Figure 4A). To further confirm this, cells were treated with 2 μM BDP5290, a potent inhibitor of MRCKα (28), and barrier integrity was evaluated by TEER. Consistent with siRNA silencing, baseline TEER was decreased upon MRCKα inhibition. More importantly, inhibitor treatment prevented the β1 subunit–induced increase of barrier integrity (Figure 4B). Immunofluorescence staining also confirmed that the β1 subunit increased intensity and membrane localization of zo-1. Again, this phenomenon was abolished when MRCKα was knocked down (Figure 4C). Collectively, our data indicate a critical role of the β1 subunit in improving alveolar barrier function through activation of MRCKα. We next tested whether overexpressing MRCKα directly was able to enhance alveolar barrier function. After ATII cells were transfected with MRCKα plasmids, TEER was measured to monitor barrier function. Twenty-four hours after transfection, we detected no significant differences in TEER; however, at both 48 and 72 hours after transfection, we observed significantly higher resistance in cells transfected with MRCKα compared with those transfected with empty plasmid (Figure 4D). These results demonstrate that overexpression of MRCKα alone is sufficient to promote alveolar epithelial barrier integrity.

### Activation of non–muscle myosin II mediates β1 subunit stabilization of tight junctions.

**Figure 5:**
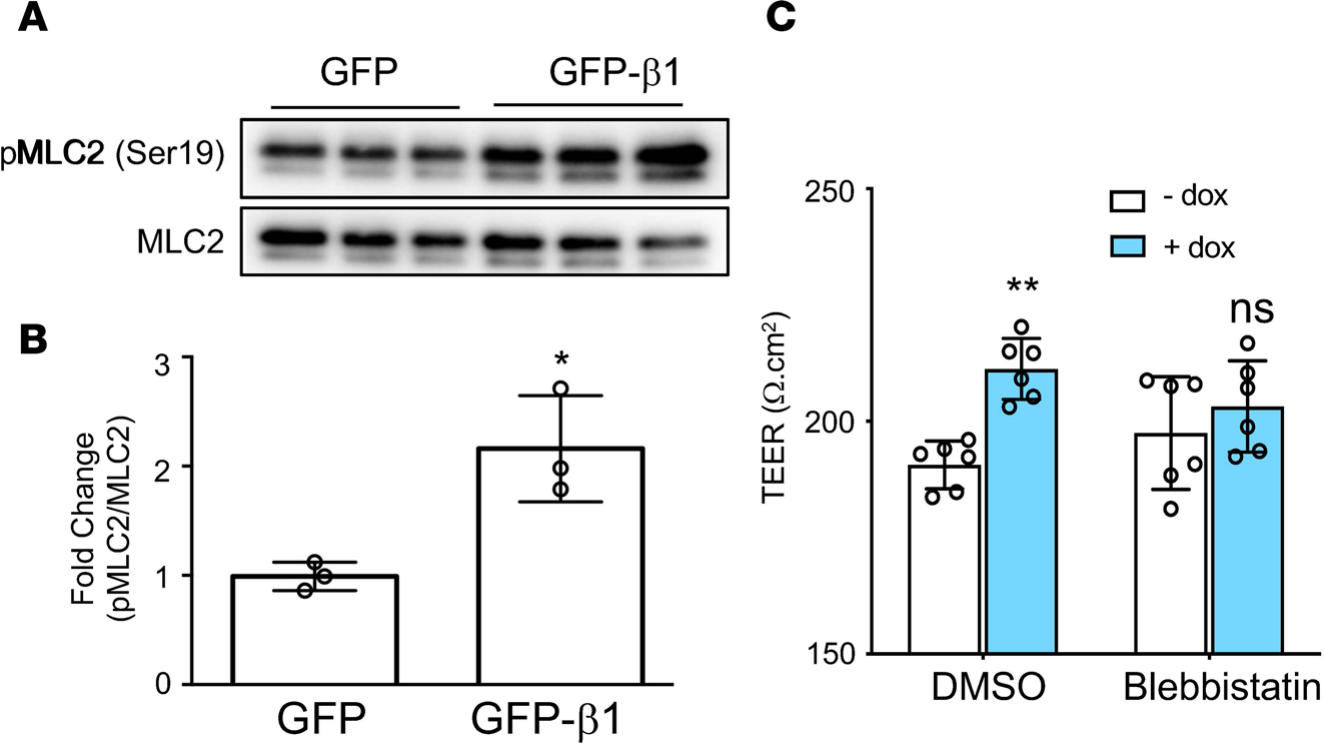
The MRCKα downstream pathway is activated upon overexpression of the β1 subunit. (**A**) Cells were electroporated with plasmid expressing the rat β1 subunit or pCDNA3 empty plasmid as control at day 3 after isolation. (**B**) Cells were lysed for Western blot 24 hours later and quantified. Data represent *n* = 3 biological replicates. Statistical analysis is by Student’s *t* test, *P < 0.05. (**C**) At day 1 after isolation, cells were cotransfected with pCMV-tet and pTet-β1 and treated with 20 μM Blebbistatin or DMSO as control. After another 24 hours, 1 μg/mL doxycycline was added to induce gene expression. TEER was measured 24 hours later. ANOVA followed by Bonferroni’s post hoc test was used for statistical analysis. **P < 0.001.

Our results support the hypothesis that the β1 subunit interacting protein MRCKα is both necessary and sufficient to promote the formation of alveolar epithelial cell tight junctions. To further substantiate this conclusion, we examined the activation of MLC2, a downstream effector of MRCKα (23). Western blot showed that overexpression of the β1 subunit induced the phosphorylation of MLC2 at Ser19 by 2-fold (Figure 5, A and B). The activation of actin-myosin via MLC2 regulates the assembly of tight junction complexes and their steady state level through endocytic degradation (29–31). In addition, the activation of MLC2 is associated with junctional recruitment, formation of circumferential actin bundles, and barrier maturation (23, 26, 27, 32, 33). Therefore, we investigated whether β1 subunit–mediated activation of MLC2 is responsible for the increased barrier integrity seen upon β1 overexpression. Pretreatment of cells with 20 μM blebbistatin, a specific inhibitor of MLC2, prevented the increase in TEER induced by overexpression of the β1 subunit (Figure 5C). Taken together, these results suggest that the activation of MLC2 is required for β1-mediated tight junction stabilization and alveolar epithelial barrier potentiation.

### Human ARDS patients show decreased expression of MRCKα.

**Figure 6:**
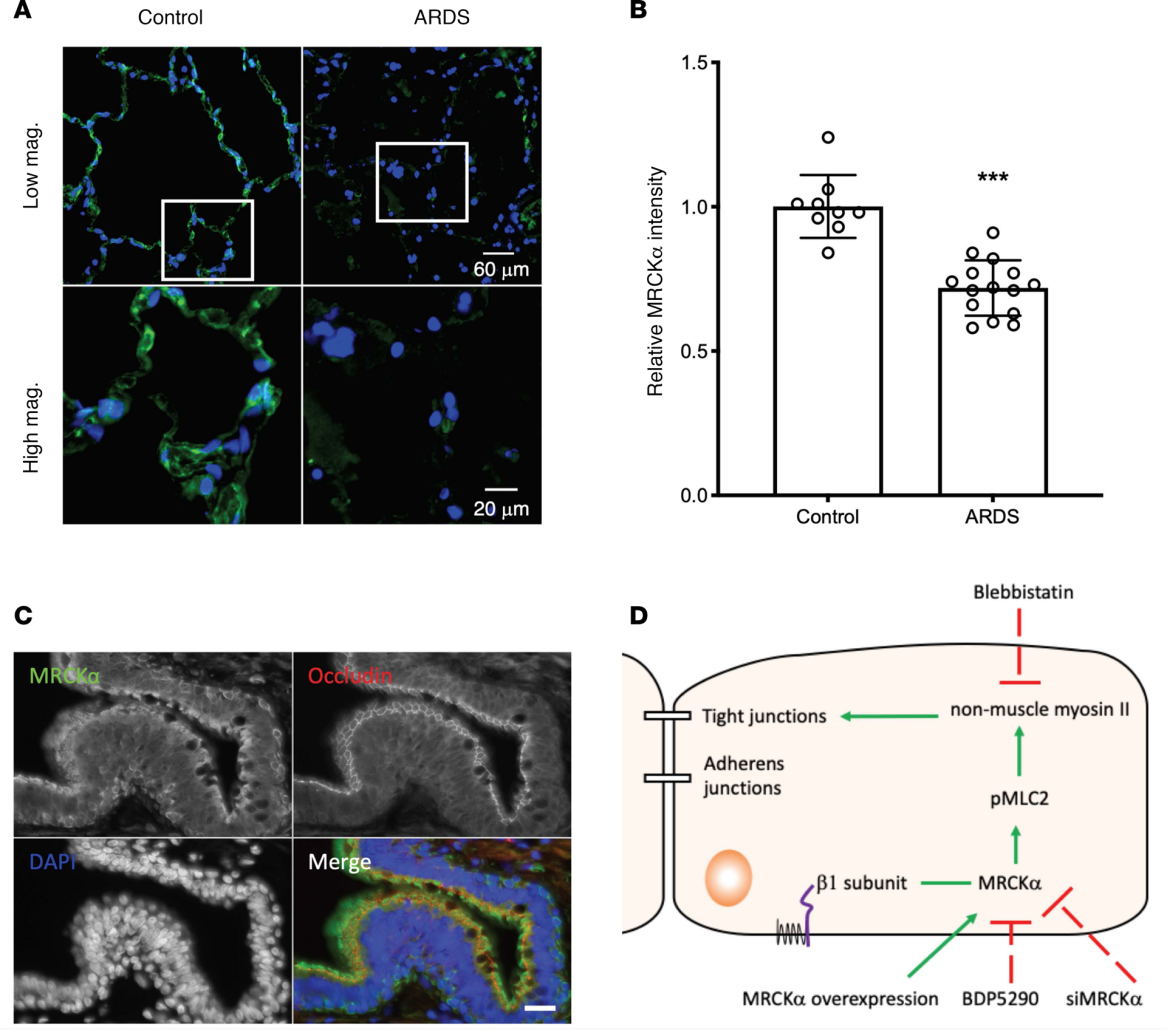
Decreased MRCKα levels in the alveolar epithelium of human ARDS patients. (**A**) Representative images of immunofluorescence staining for MRCKα (green) in lung sections of a control donor and a patient with ARDS. Upper panel shows images taken at 20× objective magnification, and lower panel shows images taken at 63× objective magnification for the boxed region in the upper panel. (**B**) Quantification of MRCKα expression in the alveoli. ROI (region of interest) were drawn in the alveoli region, and the ratio of integrated pixel intensity for MRCKα and DAPI was calculated for each ROI. Three normal donors and 5 ARDS patients were used for quantification, with 3 random fields chosen for each sample. Data are expressed as mean ± SEM, with *n* = 9 (3 patients) for normal control and *n* = 15 (5 patients) for ARDS. Statistical analysis was by 2-tailed Student’s *t* test, ***P < 0.001. (**C**) Costaining of MRCKα (green) and occludin (red) in the small airway from control donor. Scale bar: 20 µm. (**D**) Working model of the β1 subunit increases alveolar epithelial barrier integrity. The β1 subunit of the NKA interacts with MRCKα, assists in its activation, leads to higher myosin phosphorylation, and eventually stabilize tight junctions.

Given that MRCKα regulates alveolar barrier integrity, we investigated whether its expression altered in ARDS. Immunofluorescence staining demonstrated that lungs from ARDS patients (*n* = 5) express much lower levels of MRCKα compared with lungs from control donors (*n* = 3) (Supplemental Figure 5A and Figure 6A), with an average of 30% less relative fluorescent staining intensities (Figure 6B). In addition to alveoli, small airways also express high levels of MRCKα, especially in the cilia where apical occludin is expressed, and in basal cells (Figure 6C). Importantly, staining intensities in these tissues are also decreased in ARDS patients (Supplemental Figure 5B). Taken together, these data imply that lower levels of MRCKα in the lung may be associated with ARDS pathology.

## Discussion

NKA is well known for its transport activity — moving Na^+^ out of the cell and importing K^+^. Our results have identified a potentially new function of this enzyme. Specifically, we have found that the small, non–catalytic β1 subunit promotes alveolar epithelial barrier integrity through a transport-independent mechanism that involves protein interaction and activation of MRCKα (Figure 6D). Inhibition of MRCKα using either siRNA or pharmacological inhibitors prevents the upregulation of occludin and the increase of TEER induced by β1 subunit overexpression; on the other hand, overexpression MRCKα alone is sufficient to enhance barrier function. Consistent with an activation of MRCKα, overexpression of the β1 subunit increases the phosphorylation of MLC2 at Ser19 (25). Blebbistatin, a specific inhibitor of myosin II, abrogates the increase of TEER by β1 subunit overexpression. Together, these data demonstrate that the β1 subunit increases epithelial tight junction function by controlling MRCKα activation and myosin-actin activity.

During our investigation to decipher the signaling pathway, we have established a cellular model of the alveolar epithelial barrier using ATI-like cells that enables efficient and dose-dependent induction of gene expression. Using this model, we demonstrated that overexpression the β1 subunit leads to improved barrier integrity, as demonstrated by the upregulation of tight junction proteins occludin, zo-1, zo-2, and claudin-18; increased electrical resistance; and decreased permeability to fluorescent tracers. This study supplements previous findings in mice and pigs (11, 13, 34), and it provides a mechanistic basis to apply ARDS gene therapy approaches for potential human clinical use. The cellular model that we established here can also be used to study other lung diseases characterized by barrier defects, such as asthma. Importantly, claudin-18 is the most abundant claudin in ATI cells (14). Deficiency in claudin-18 results in both alveolar and airway barrier dysfunction (14–16). However, the claudin-18–KO mouse has increased levels of the NKA β1 subunit but decreased occludin expression (15). This suggests a possible compensatory effect between the β1 subunit and claudin-18 during development. Our unpublished data also suggest a functional interaction between occludin and claudin-18, indicating that the proper expression and localization of tight junction proteins may depend on each other.

Our data suggest a β1 subunit–specific effect in regulating alveolar barrier integrity among all NKA β subunits. Surprisingly, overexpression of the β3 subunit, but not the β2 subunit, decreased expression of the β1 subunit, occludin, and zo-1 (Supplemental Figure 2, A and B). It is worth mentioning that such a competing mechanism between β1 and β3 subunits, but not with the β2 subunit, has been reported previously in the literature (35). β1 Subunit–KO mice show higher β3 subunit expression (36). However, overexpression of the β2 subunit in WT mice did not decrease β1 subunit levels (37). Future experiments to compare the effect of the 3 subunits in treating LPS-induced lung injury will further substantiate our findings. The conclusion that β1 subunit–mediated tight junction upregulation is a process independent of the ion transport activity of the NKA is consistent with previous findings from our laboratory (10, 11, 13) and others (7–9, 38) that only the β1 subunit, but not the α subunit or the epithelial sodium channel, decreases lung permeability and treats mice with existing ARDS.

Our results have confirmed some known protein interactions of the β1 subunit, including the NKA α1 subunit, the ER protein Wolframin (39), coatomer subunit β (40), and lethal giant larvae protein (41). Some proteins previously reported to interact with the β1 subunit (42–45) were not detected in our analysis, likely because these proteins — which are mainly expressed in the neural system — are not expressed in the lung. More importantly, many binding partners have been identified. To our knowledge, this is the first proteomic analysis of the β1 subunit interaction network. The interactome of many integral membrane proteins has remained unknown or is only poorly characterized due to their hydrophobicity, low expression, and lack of trypsin cleavage sites in their transmembrane segments (46, 47). The current MS analysis greatly enriches our knowledge of the protein interactome of the β1 subunit. The binding partners identified from this study can be confirmed by future experiments and will provide important insight regarding the activity and cellular functions of NKA.

A previous study using siRNA injection into mouse embryos proposed that the β1 subunit is required for proper distribution of tight junctions, likely via regulation of the actin cytoskeleton (48). Our data suggest that MRCKα appears to be involved in these processes. MRCKα is involved in cell migration, polarization, and junction formation by regulating actin-myosin activity (23, 24, 26). In accordance with our finding, a previous study in endothelial cells suggests that MRCKα mediates the activation of non–muscle myosin at cell-to-cell contacts and the formation of circumferential actin bundles, which is essential for cell junctions (23). The mechanism of how MRCKα is activated upon interacting with the β1 subunit is unknown. It is possible that the interaction promotes the plasma membrane localization of MRCKα, similar to that seen for the NKA β1 subunit and the sodium calcium exchanger 1 (49) or megalencephalic leukoencephalopathy with subcortical cysts 1 (44). Another possibility is that the β1 association with MRCKα abolishes the autoinhibition of MRCKα by binding to its 2 distal CC domains, which interact intramolecularly with the kinase domain and negatively regulate its activity (21). These 2 events may also happen concurrently. Future investigation is needed to test these possibilities.

One striking finding from our results is that lungs from patients with ARDS tend to express lower amounts of MRCKα. No genetic susceptibility of ARDS has been linked to MRCKα so far. However, one of its downstream targets, MLC kinase, is associated with ARDS susceptibility and outcomes (50). Additionally, a recent study suggested that MRCKα is involved in epithelial extrusion following apoptosis (51). Epithelial extrusion is a process by which dying or unwanted cells are removed from an epithelium while preserving the barrier function of the layer (52). To date, no study has explored the physiological and pathological roles of MRCKα in the lung. It will be interesting to investigate whether decreased MRCKα results in a defect of epithelial extrusion, thereby predisposing the lung to injuries that ultimately lead to ARDS.

The reason lungs from ARDS patients express significantly lower amounts of MRCKα is unclear. One possibility is lower basal transcription of MRCKα due to genetic causes (such as reduced gene copy numbers or epigenetic modification). Another possibility is that risk factors for ARDS, such as inflammation, may downregulate MRCKα levels. Regardless, MRCKα could be a drug target for treating ARDS or other human diseases characterized by barrier defects. Currently, only inhibitors of MRCKα have been identified (28, 53). Activation of MRCKα may be achieved by using a peptide that corresponds to the interacting domains on the NKA β1 subunit. Such a peptide modulator could be a promising drug to enhance epithelial barrier function and could ultimately lead to a simple pharmacological treatment of ARDS.

In conclusion, our data have supported a nontransport associated role of the NKA β1 subunit in the regulation of tight junctions. This work enhances our understanding of the NKA and defines a role for MRCKα in the homeostasis of lung epithelial barrier properties.

## Methods

### Plasmids and siRNA.

pCDNA3 and pCMV-EGFP plasmids were purchased from Invitrogen. Mouse NKA β2 subunit and mouse NKA β3 subunit with Myc-DDK tag were obtained from OriGene. The Tet-On 3G drug-inducible gene expression system was purchased from Clontech. The human NKA β1 subunit–coding sequence was inserted into the pTRE3G vector at the SalI and BamHI restriction enzyme sites. The human MRCKα plasmid was a gift from Paolo Armando Gagliardi at the University of Bern (Bern, Switzerland) (24). Knockdown was carried out using the TriFECTa DsiRNA Kit (IDT) according to manufacturer’s instructions. siRNA duplexes at a final concentration of 100 nM were transfected in 4 mm cuvettes (Bio-Rad) using a GenePulser Xcell (Bio-Rad) instrument.

### Antibodies and inhibitors.

Primary antibodies for Western blot include anti–NKA β1 subunit (Upstate, 05-382), anti-occludin (Invitrogen, 71-1500), anti–zo-1 (Invitrogen, 61-7300), anti–zo-2 (Invitrogen, 71-1400), anti-actin (MilliporeSigma, A2066), anti-GAPDH (MilliporeSigma, CB1-001), anti–NKA β2 subunit (Abcam, ab185210), anti-DDK (OriGene, TA50011-100), anti-MRCKα (Santa Cruz Biotechnology Inc., sc-374568), anti-MYPT1 (Cell Signaling Technology, 2634S), anti–phospho-MYPT1 (Thr696, Cell Signaling Technology, 5163S), anti-MLC2 (Cell Signaling Technology, 3672S), and anti–phospho-MLC2 (Ser19, Cell Signaling Technology, 3671S). The primary antibodies for immunofluorescence include anti–occludin Alexa Fluor594 (Invitrogen, 331594), anti–zo-1-Alexa Fluor594 (Invitrogen, 339194), and anti-MRCKα (Thermo Fisher Scientific, PA1-10038). The inhibitor for MRCKα BDP5290 was purchased from Aobious. Myosin inhibitor blebbistatin was purchased from Abcam.

### Primary cell isolation and cell culture.

Primary rat ATII cells were isolated using an IgG-panning approach as described by Dobbs et al. (54). Briefly, lungs from Sprague Dawley rats (Charles River Laboratories) (200–250 g) were surgically removed and perfused, lavaged, and treated with 1 mg/mL elastase (Worthington Biochemical) to release the epithelial cells. Next, lung lobes were separated, cut, minced, filtered, and spun down at 1500 rpm for 15 minutes. The cells were resuspended in DMEM without FBS and transferred to 2 IgG plates. After incubation at 37˚C for 1 hour, nonadhered cells (predominately ATII cells) were transferred to a new tube and centrifuged at 250*g* at room temperature for 15 minutes. The cells were resuspended in DMEM containing 10% FBS and plated on fibronectin coated plates. To coat the plates with fibronectin, 20 μg/mL fibronectin from bovine plasma (F1141, MilliporeSigma) was added to 100 mm culture plates (using 3 mL) or the upper chamber of the transwell plates (using 400 μL/well). Plates were left at 37°C for 3 hours. Residual solution was removed, and plates were dried in a tissue culture hood for at least 30 minutes before cells were added. 16HBE14o- human bronchial epithelial cells were cultured in DMEM as previously reported (13).

### Transfection.

Transfection was carried out by electroporation using the Gene Pulser MXcell electroporation system (Bio-Rad). The electroporation conditions for ATI cells was 1 square wave pulse at 300 V, 1000 Ω, and 20 milliseconds. A total of 10 μg plasmid DNA was used for 1 × 10^6^ cells.

### Western blot.

Cells were lysed with reporter lysis buffer (1×, Promega) supplemented with protease inhibitor (cOmplete, Mini, EDTA-free tablets; Roche) and phosphatase inhibitor (PhosStop Phosphatase Inhibitor Cocktail; Roche). Proteins were separated on 10% SDS-PAGE gels, transferred to PVDF membrane, and probed with primary antibodies at room temperature for 2 hours or at 4°C overnight. After incubation with secondary antibodies and development, bands were detected on film (Biomax MR film; Carestream Health) or using the ChemiDoc Imaging System (Bio-Rad) and quantified using Image Studio Lite software (Li-COR) or Image Lab software (Bio-Rad).

### Plasma membrane isolation.

HEK293 cells were transfected with plasmids expressing GFP, or plasmids expressing GFP-β1 using Lipofectamine 2000 (Invitrogen). Two days after transfection, 6 × 10^6^ cells were subjected to membrane isolation using the Minute Plasma Membrane Protein Isolation and Cell Fractionation Kit (Invent Biotechnologies), and proteins were analyzed by SDS-PAGE and Western blots.

### qPCR.

Total RNA was isolated using the RNeasy Mini Kit (Qiagen). After determining RNA concentrations by spectrophotometry, 100–1000 ng of total RNA was used for cDNA synthesis. Reverse transcription was conducted using the Reverse Transcription System (Promega). A total of 10 μL of the reaction was diluted to 100 μL, from which 1 μL was taken for qPCR using iTaq Universal SYBR Green Supermix (Bio-Rad). The specificity of primers was confirmed by melting curve analysis and gel electrophoresis. qPCR was performed on a CFX Connect Real Time PCR Detection System (Bio-Rad). Samples were assayed in triplicate. Relative RNA level was quantified using the ΔΔCt method and normalized to the endogenous control GAPDH unless specified otherwise.

### Immunofluorescence.

Cells were washed 3 times before fixation with 4% paraformaldehyde in PBS for 15 minutes at room temperature. Fixed cells were washed with PBS and permeabilized with 0.2% Triton X-100 in PBS for 10 minutes. After washing with PBS, transwell inserts were blocked with blocking reagent (Dako Protein Block Serum Free, Agilent) for 1 hour and incubated with primary antibody at 4°C overnight. Nuclei were stained with 2.5 μg/mL DAPI for 5 minutes and then washed twice with PBS. The transwell membrane was then carefully cut out using a clean razor blade and mounted on a glass slide with ProLong antifade mounting media (Thermo Fisher Scientific). Slides were examined under a Leica DMI6000 microscope, and photos were captured using the open source software μManager or Volocity software (Velocity Inc.). Tissue sections of human lungs from patients with ARDS were provided by Zhongren Zhou in the Department of Pathology at the University of Rochester using an IRB-approved protocol. All samples were taken at autopsy. In total, 16 sections from 6 ARDS patients and 7 sections from 3 control patients without ARDS were obtained. The H&E staining of each corresponding section shows varying degrees of lung injury and edema content. For immunofluorescence staining, tissue sections were deparaffinized and rehydrated. Then, an antigen retrieval step was performed to expose epitopes for subsequent antibody binding and immunofluorescence.

### TEER.

Prior to measuring TEER, cells cultured on 12-well transwell plates (12 mm transwell with 0.4 μm pore polyester membrane insert; Corning) were moved to the tissue culture hood for 15 minutes to allow the medium to equilibrate to room temperature. TEER was measured using an epithelial voltmeter (EVOM2; World Precision Instruments). Three to 6 wells were measured for each condition, and 3 readings were recorded and averaged for each well. To calculate TEER, the resistance of the fibronectin-coated insert without cells (blank resistance) was subtracted from the measured resistance and then multiplied by 1.12 cm^2^ to account for the surface area of the insert.

### Permeability.

Permeability to fluorescent tracers was measured using a modified protocol previously described (55). After TEER measurement, the upper and lower transwell chamber were washed twice with P buffer (10 mM HEPES at pH 7.4, 1 mM sodium pyruvate, 10 mM glucose, 3 mM CaCl_2_, and 145 mM NaCl) (Invitrogen). A total of 500 μL of freshly prepared solution containing 100 μg/mL of 40 kD FITC-dextran and 100 μg/mL of 3kD Texas Red–dextran was added to the apical compartment. A total of 1000 μL of P buffer was added to the bottom chamber. After 2 hours incubation at 37°C, 100 μL of the basal medium was collected, and the fluorescence of the transported dextran was measured with a SpectraMax M5 multimode microplate reader (Molecular Devices). The excitation wavelength and emission wavelength are 492 nm and 520 nm for FITC and 596 nm and 615 nm for Texas-red, respectively. The quantity of tracer was calculated by comparison with a standard curve. A permeability coefficient was determined using the following equation (56): Pc (cm/min) = V/(A × Co) × (C/T), where V is volume in the lower compartment (1 mL), A is the surface area of the membrane (1.12 cm^2^ for the 12-well transwell used here), Co is the dextran concentration in the upper compartment at time 0 (0.1 mg/mL), and C is the dextran concentration in the lower compartment at time T of sampling (2 hours).

### IP and MS.

Cells from one 100 mm plate were lysed with 1 mL of IP lysis buffer (1% NP-40, 50 mM Tris HCl at pH 8.0) and homogenized 10 times with a 25-gauge syringe. IP was performed using the μMACS Protein G Kit according to the manufacturer’s instructions (Miltenyi Biotec). The precleared samples were incubated with anti-MRCKα antibody (PA1-10038, 1:50 dilution; Thermo Fisher Scientific), anti-β1 antibody (Upstate, 05-382, 1:250 dilution), or IgG as control at 4°C overnight. The elute was analyzed by SDS-PAGE Gradient Gels (4%–20%). Each lane was cut into 10 pieces of approximately the same size. The gel bands were then destained, reduced, and digested with trypsin overnight. The digested peptide mixtures were then subjected to LC-MS/MS analysis using the Orbitrap system.

### Label-free quantification of proteins interacting with the β1 subunit.

Thermo raw data were transformed into mgf format. The resulting peak lists were searched using Protein Prospector (v5.22.0) with the following settings: Trypsin as protease with a maximum of 1 missed cleavage site, 10 ppm mass tolerance for MS, 0.5 Da (ion trap), and 0.05 Da (Orbitrap), respectively, for MS/MS, carbamidomethylation (C) as fixed, oxidation (M), and phosphorylation (S/T/Y) as variable modifications. Results from Protein Prospector were retrieved and cleaned up using in-house python script. Protein quantitation using the NSAF measurement was described previously (19). Data normalization, annotation, and statistical analysis were performed using Perseus (57). Two-tailed Student’s *t* test was used for statistical analysis of NSAF (58). The proteomics data have been deposited to the ProteomeXchange Consortium via MassIVE (Mass Spectrometry lnteractive Virtual Environment) with the accession no. MSV000084881.

### Statistics.

Each experiment was repeated at least 3 times. The data of each series is displayed as mean values ± SD unless otherwise noted. Graphing and statistical comparison of the data were performed using Prism 7 (GraphPad Software). Measurements for 2 groups were analyzed using the Student’s *t* test. Measurements for more than 2 groups were analyzed by 1-way ANOVA and multiple comparisons. *P* values less than 0.05 were considered to be statistically significant.

### Study approval.

All animal studies were approved by the University of Rochester Committee on Animal Resources, and experimental procedures were carried out under the institutional guidelines for the care and use of laboratory animals in an American Association for the Accreditation of Laboratory Animal Care–approved facility. Human lung tissues from patients with and without ARDS was obtained at autopsy at the University of Rochester using and IRB approved protocol.

## Author contributions

HB and DAD conceived, designed, and analyzed experiments and wrote the manuscript. HB and RZ performed experiments. MB, RN, and RZ performed primary cell isolation. AF assisted in MS. DY was involved in staining of human tissues. XL and JLY advised and conceived experiments.

## Supplementary Material

Supplemental data

## Figures and Tables

**Table 1:**
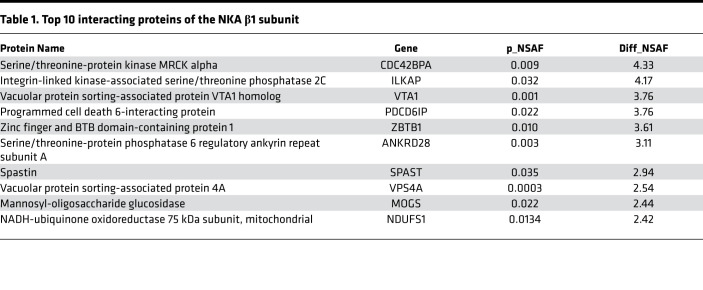
Top 10 interacting proteins of the NKA β1 subunit

**Table 2:**
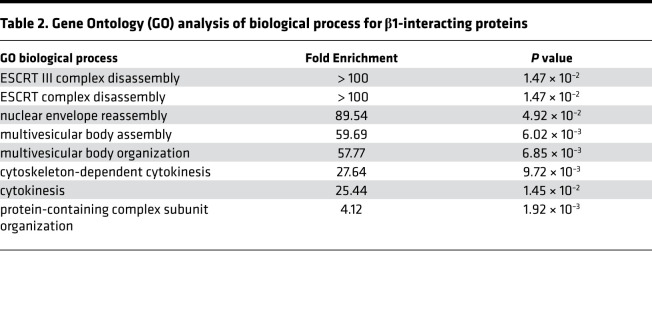
Gene Ontology (GO) analysis of biological process for β1-interacting proteins

## Version 1 01/28/2021

In-Press Preview

## Version 2 02/22/2021

Electronic publication
