# Supplementary material for: The Na+, K+-ATPase β1 subunit regulates epithelial tight junctions via MRCKα
Source: JCI Insight. 2021 Feb 22;6(4):e134881. doi: 10.1172/jci.insight.134881 (PMC7934944; doi:10.1172/jci.insight.134881)

Figure S1. Characterization of ATII to ATI cell differnetiation. (A) ATI cells were cultured on a 12-well transwell plate coated with 20 mg/ml fibronectin or PBS as control. TEER was measured every 24 hours after plating (n=4 for each group). **(B)** After day 4, cells were fixed and stained for occludin or zo-1 (red) and counterstained with DAPI (blue). Images were taken at 20x. **(C)** At day 3 after isolation when cells became confluent and displayed an ATI phenotype, 1 μg/ml LPS was added and TEER was measured 24 hours later. **(D)** Following measurement of TEER in C, permeability to 3kD FITC-dextran was assayed. *$p<0.05$; **$p<0.01$. **(E).** Transfection of the β1 subunit also increases other tight junction proteins, including zo-2 and claudin-18. AT1 cells (n=3) were transfected with pCMV-EGFP or pCMV-GFPβ1 plasmids and lused for Western blots 24 hours later.

Figure S2. The β1 subunit mediated upregulation of tight junction proteins is ion-transport independent. (A) ATI cells (day 3 after isolation) were transfected with plasmid expressing the mouse β2 subunit or pCDNA3 empty plasmid as control. Cells were lysed for western blot analysis after 24 hours. **(B)** ATI cells (day 3 after isolation) were transfected with plasmid expressing the mouse β3 subunit containing a DDK tag or pCDNA3 empty plasmid as control. Cells were lysed for western blot analysis after 24 hours.

Figure S3. Dose-dependent induction of β1 subunit gene expression using a Tet-on system. (A) 16HBE14o- cells were cotransfected with pCMV-tet regulator plasmids and pTet3G-human β1 subunit expressing plasmids by electroporation, followed immediately by addition of 0, 1, 10, 100, and 1000 ng/ml doxycycline. Cells were lysed for western blot analysis 24 hours after electroporation. **(B)** 16HBE14o- cells were cotransfected with pCMV-tet plasmids and tet-

lucifersase plasmids by electroporation. After transfection, cells were treated with 1 μg/ml doxycycline or H_2O as control. Wells of cells (n=3) were lysed with reporter lysis buffer every other day and luminescence was measured. RLU: relative luminescence unit. **(C)** qPCR for *SPC* and *CAV1* shows that dox treatment does not affect the differentiation from ATII to ATI.

Figure S4. Identifying MRCKα as an interacting protein with the β1 subunit. (A) SDS-PAGE of immunoprecipitation using antibody against the β1 subunit or GFP antibody as control **(B)** Amino acid coverage of MRCKα. Peptides identified by mass spectrometry are marked in red.

Figure S5. Decreased MRCKα level in lung sections from human ARDS patients. (A). Staining of MRCKα from sections of 3 normal control donors and 6 ARDS patients. Three random fields were chosen for each patient for intensity quantitation. Images from ARDS patient #5 were excluded from analysis due to high background signal. **(B)** Representative staining of MRCKα in the airways of control donor and ARDS patient.

Table S1. Number of proteins identified from triplicate MS experiment

Repeat	Control	β1
1	1253	1322
2	1081	1204
3	1325	1581

Figure S1

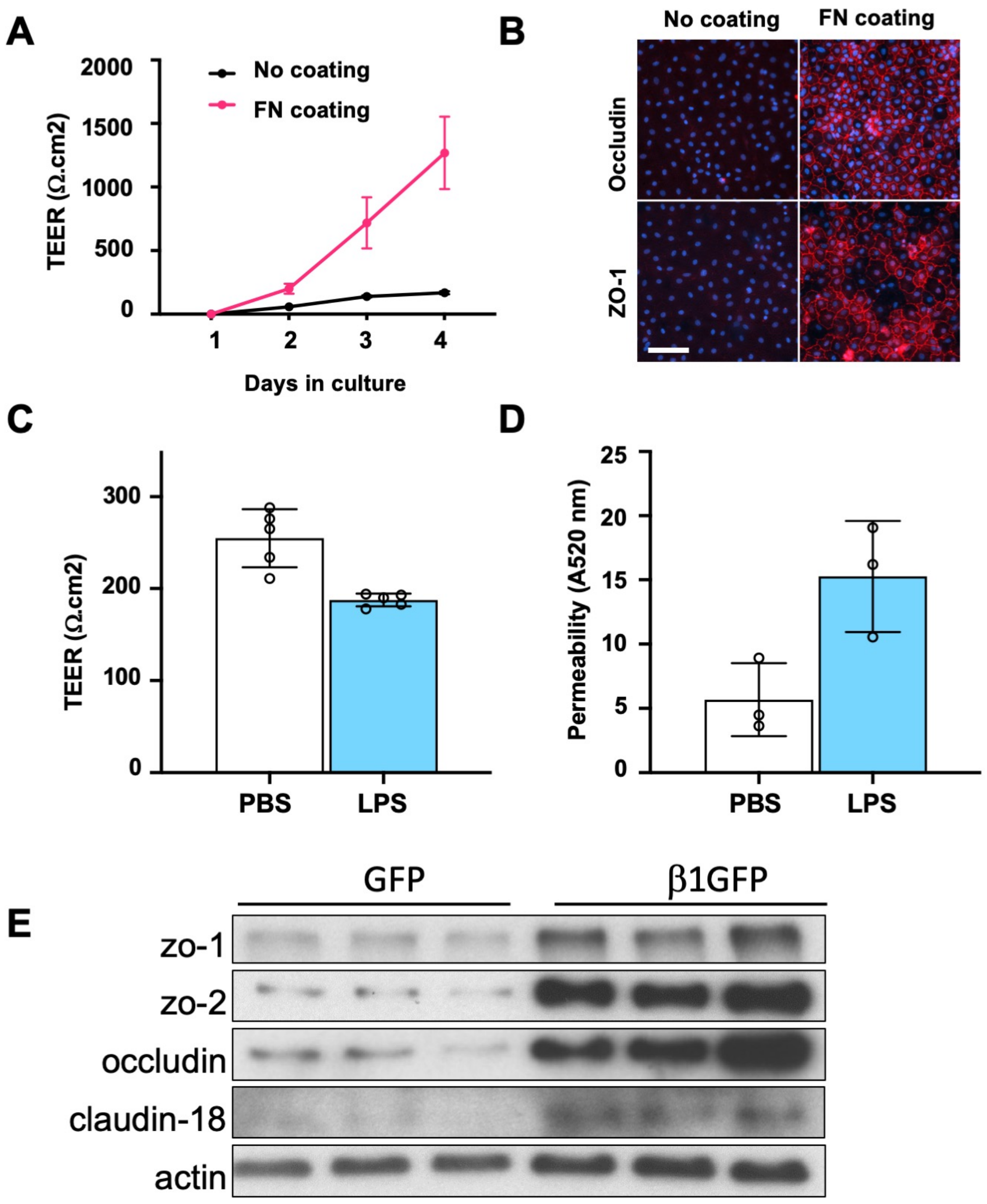
A
2000
1500
1000
500
0
TEER (Ω.cm2)
No coating
FN coating
1
2
3
4
Days in culture
B
No coating
FN coating
Occludin
ZO-1
C
300
200
100
0
TEER (Ω.cm2)
PBS
LPS
D
25
20
15
10
5
0
Permeability (A520 nm)
PBS
LPS
E
GFP
β1GFP
zo-1
zo-2
occludin
claudin-18
actin

Figure S2

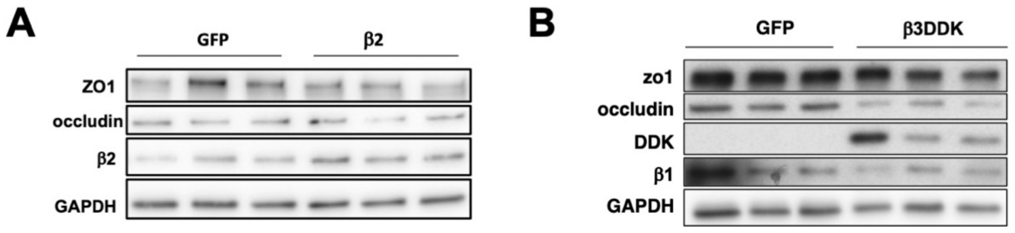
A
GFP
β2
ZO1
occludin
β2
GAPDH
B
GFP
β3DDK
zo1
occludin
DDK
β1
GAPDH

Figure S3

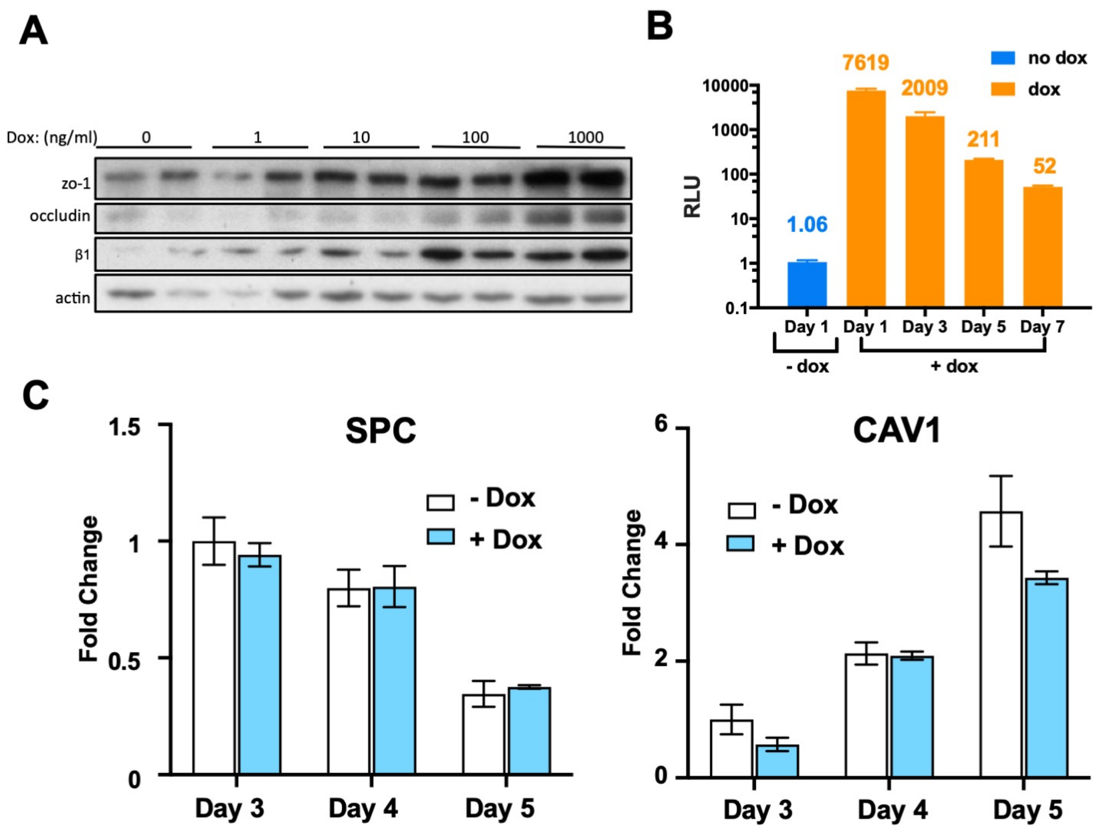
A
Dox: (ng/ml)
0
1
10
100
1000
zo-1
occludin
β1
actin
B
no dox
dox
7619
2009
211
52
1.06
RLU
10000
1000
100
10
1
0.1
Day 1
Day 1
Day 3
Day 5
Day 7
- dox
+ dox
C
SPC
- Dox
+ Dox
Fold Change
1.5
1
0.5
0
Day 3
Day 4
Day 5
CAV1
- Dox
+ Dox
Fold Change
6
4
2
0
Day 3
Day 4
Day 5

Figure S4

A

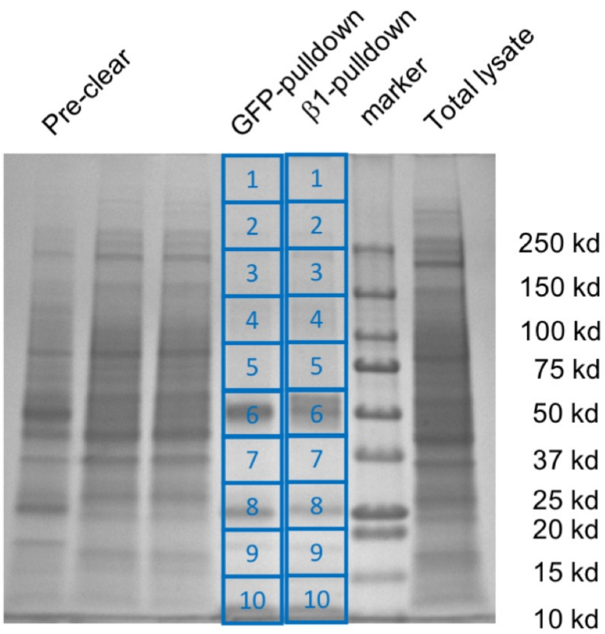

B

1 MSGEVRLRQL EQFILDGPAQ TNGQCFSVET LLDILICLYD ECNNSPLRRE KNILEYLEWA KPFTSKVKQM R**LHREDFEIL**
81 **K**VIGRGAFGE VAVVKLKNAD KVFAMK**ILNK WEMLK**RAETA CFREER**DVLV NGDNKWITTL HYAFQDDNNL YLVMDYYVGG**
161 **DLLTLLSKFE DRLPEDMAR**F YLAEMVIAID SVHQLHYVHR DIKPDNILMD MNGHIRLADF GSCLKLMEDG TVQSSVAVGT
241 PDYISPEILQ AMEDGKGRYG PECDWWSLGV CMYEMLYGET PFYAESLVET YGKIMNHKER **FQFPAQVTDV SENAK**DLIRR
321 **LICSREHR**LG QNGIEDFKK**H PFFSGIDWDN IR**NCEAPYIP EVSSPTDTSN FDVDDDCLKN SETMPPPTHT AFSGHHLPFV
401 GFTYTSSCVL SDRSCLR**VTA GPTSLDLDVN VQRTLDNNLA TEAYER**RIKR **LEQEKLELSR KLQESTQTVQ ALQYSTVDGP**
481 **LTASKDLEIK NLKEEIEKLR KQVTESSHLE QQLEEANAVR** QELDDAFRQI KAYEKQIK**TL QQEREDLNKE LVQASER**LKN
561 QSKELKDAHC QR**KLAMQEFM EINER**LTELH TQKQKLARHV R**DKEEEVDLV MQK**VESLRQE LRRTERAK**KE LEVHTEALAA**
641 **EASKDR**K**LRE QSEHYSKQLE NELEGLKQK**Q ISYSPGVCSI EHQQEITK**LK TDLEK**K**SIFY EEELSKREGI HANEIK**NLKK
721 **ELHDSEGQQL ALNKEIMILK DK**LEKTRR**ES QSEREEFESE FK**QQYEREK**V LLTEENKKLT SELDK**LTTLY ENLSIHNQQL
801 EEEVKDLADK KESVAHWEAQ ITEIIQWVSD EK**DARGYLQA LASKMTEELE ALR**NSSLGTR **ATDMPWK**MRR **FAKLDMSARL**
881 **ELQSALDAEI R**AK**QAIQEEL NK**VK**ASNIIT ECKLKDSEKK NLELLSEIEQ LIK**DTEELRS EKGIEHQDSQ HSFLAFLNTP
961 TDALDQFER**S PSCTPASKGR** R**TVDSTPLSV HTPTLR**KKGC PGSTGFPPKR K**THQFFVKSF TTPTK**CHQCT SLMVGLIRQG
1041 CSCEVCGFSC HITCVNKAPT TCPVPPEQTK GPLGIDPQK**G IGTAYEGHVR** IPKPAGVKKG WQRALAIVCD FK**LFLYDIAE**
1121 **GK**ASQPSVVI SQVIDMR**DEE FSVSSVLASD VIHASR**KDIP CIFR**VTASQL SASNNK**CSIL MLADTENEKN K**WVGVLSELH**
1201 **KILK**KNKFRD RSVYVPK**EAY DSTLPLIKTT QAAAIIDHER IALGNEEGLF VVHVTK**DEII RVGDNKK**IHQ IELIPNDQLV**
1281 **AVISGRNR**HV R**LFPMSALDG RETDFYKLSE TKGCQTVTSG K**VRHGALTCL CVAMKR**QVLC YELFQSKTR**H RKFKEIQVPY
1361 NVQWMAIFSE QLCVGFQSGF LRYPLNGEGN PYSMLHSNDH TLSFIAHQPM DAICAVEISS KEYLLCFNSI GIYTDCQGRR
1441 SRQQELMWPA NPSSCCYNAP YLSVYSENAV DIFDVNSMEW IQTLPLKK**VR PLNNEGSLNL LGLETIR**LIY FKNK**MAEGDE**
1521 **LVVPETSDNS R**KQMVRNINN KRRYSFRVPE EERMQQRREM LRDPEMRNK**L ISNPTNFNHI AHMGPGDGIQ ILKDLPMNPR**
1601 **PQESRTVFSG SVSIPSITKS RPEPGRSMSA SSGLSAR**SSA QNGSALK**REF SGGSYSAK**R**Q PMPSPSEGSL SSGGMDQGSD**
1681 **APARDFDGED SDSPR**HSTAS NSSNLSSPPS PASPRKTK**SL SLESTDRGSW DP**

Figure S5

A

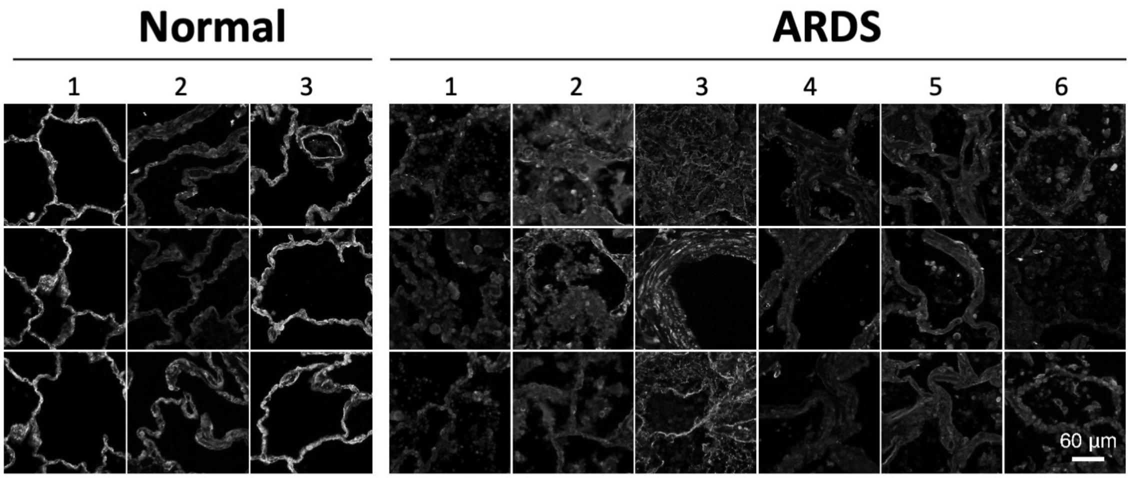

B

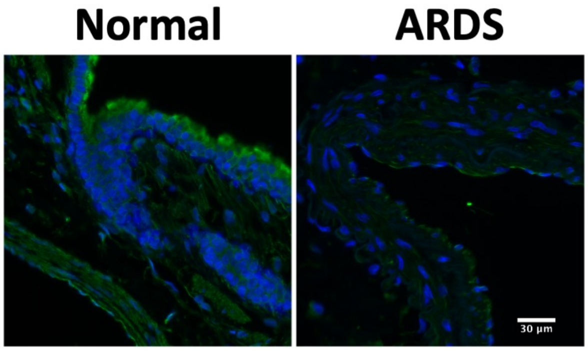

Supplement: Supplemental data [file jciinsight-6-134881-s215.pdf]
